# Pregnancy diabetes: A comparison of diagnostic protocols based on point-of-care, routine and optimized laboratory conditions

**DOI:** 10.1038/srep16302

**Published:** 2015-11-06

**Authors:** Sjoerd A. A. van den Berg, Monique J. M. de Groot, Lorenzo P. W. Salden, Patrick J. G. J. Draad, Ineke M. Dijkstra, Simone Lunshof, Sjoerd W. van Thiel, Kristel J. M. Boonen, Marc H. M. Thelen

**Affiliations:** 1 Dept. of Clinical Chemistry and Hematology, Amphia Hospital, Breda, The Netherlands; 2 Dept. of Clinical Chemistry, Sint Elisabeth Hospital, Tilburg, The Netherlands; 3 Dept. of Clinical Chemistry, Sint Anthonius Hospital, Nieuwegein, The Netherlands; 4 Dept. of Gynaecology, Amphia Hospital, Breda, The Netherlands; 5 Dept. of Internal Medicine, Amphia Hospital, Breda, The Netherlands

## Abstract

*In vitro* glycolysis poses a problem during diabetes screening, especially in remote laboratories. Point-of-care analysis (POC) may provide an alternative. We compared POC, routine and STAT analysis and a feasible protocol during glucose tolerance test (GTT) for pregnancy diabetes (GDM) screening. In the routine protocol, heparin tubes were used and turn-around-time (TAT) was unsupervised. In the STAT protocol, tubes were processed immediately. The feasible protocol comprised of citrated tubes with a TAT of 1 hour. Outcome was defined as glucose concentration and clinical diagnosis. Glucose measured by POC was higher compared to routine analysis at t = 0 (0.25 mM) and t = 120 (1.17 mM) resulting in 17% more GDM diagnoses. Compared to STAT analysis, POC glucose was also higher, although less pronounced (0.06 and 0.9 mM at t = 0 and t = 120 minutes, respectively) and misclassification was only 2%. Glucose levels and clinical diagnosis were similar using the feasible protocol and STAT analysis (0.03 mM and −0.07 mM at t = 0 and t = 120, 100% identical diagnoses). POC is an viable alternative for STAT glucose analysis in GDM screening (sensitivity: 100%, specificity: 98%). A feasible protocol (citrated phlebotomy tubes with a TAT of 60 minutes) resulted in 100% identical outcome and provides the best alternative.

Pregnancy diabetes (GDM) is associated with an increased risk of maternal, fetal and neonatal mortality and morbidity^1^. It has been described that approximately 3–5% of all pregnant women develop GDM. However, it must be recognized that it is hard to determine a true estimation of the prevalence of GDM, due to the fact that both screening and diagnostic procedures are not standardized^2^. Therefore, the local incidence may be higher. Because of the ease of use, low patient burden, and cost attractiveness, point-of-care testing (POC) has become a method of choice in a number of laboratories in the context of pregnancy diabetes screening. However, most of the POC glucose analyzers lack the accuracy of laboratory analysis^3^, possibly rendering them more useful for follow up than for diagnostic use^4^. Recently, there has been much debate on the implementation of POC analysis in the context of GDM screening. Studies give a negative advise for the use of POC based on the lack of accuracy^4^, while others argue that it could have its merits^5^,^6^, or that it should be used in concert with venous glucose determination^7^. In part, the large deviation of POC from venous measurements may be due to the difference in the sampled tissues and the differences in dynamics of the interstitial and venous compartment^8^. It has indeed been recognized that glucose kinetics are different when sampled from venous or capillary blood^9^. On the other hand, part of the disagreement between POC analysis and venous sampling may also be due to variation in the latter. This variation may largely be due to non-optimal laboratory conditions. The choice of phlebotomy material and post-phlebotomy turn-around-time (TAT) affects *in vitro* glucose stability and thus, concentration^10^ *In vitro*, glucose levels may drop as much as 7% per hour (±0.6 mM/h) due to ongoing glycolysis^11^,^12^ and secondary factors such as leukocyte count^13^ and ambient temperature may even double *in vitro* glycolytic rate.

To guarantee accurate glucose measurements, tubes containing anti-glycolytic agents are used^14^. The mode of action of the most commonly used anti-glycolytic agent, sodium fluoride (NaF), is based on the inhibition of enolase activity^15^. However, although inhibition of enolase activity stabilizes the glucose concentration in the long term, it does not prevent a drop during the first hours after phlebotomy^16^. Therefore, it is not unlikely that part of the disagreement found between POC and venous glucose concentration is due to an unstable glucose concentration in the phlebotomy tube (due to ongoing *in vitro* glycolysis), which thereby biases the debate on the usability of POC in screening for GDM. Here, we present a comparison of POC to routine laboratory analysis as well as to optimized laboratory conditions during glucose tolerance tests in pregnant women. In the routine laboratory condition protocol, TAT of the phlebotomy material was not pre-defined and thus dependent on the day-to-day and hour-to-hour variation. No measures were taken to prevent glycolysis. In the optimized laboratory condition protocol, TAT was defined to be less than 5 minutes and glycolysis was prevented by direct centrifugation, separation of cells and plasma and cryopreservation of the plasma until analysis. In addition to these studies, we have explored the feasibility of a protocol, based on citrated phlebotomy tubes with a TAT of 60 minutes, subsequent plasma analysis, and compared that to the results of the optimal laboratory protocol. Outcome was based on the similarity of laboratory results (defined as glucose concentration), as well as the agreement in clinical outcome (defined as GDM diagnosis).

## Research Design and Methods

### Subjects and phlebotomy

The study described here was conducted according to the principles of the Declaration of Helsinki, adapted in 2013 (Fortaleza, Brazil) and in accordance with the Dutch Medical Research Involving Human Subjects Act (WMO; study number NL46462.015.13). The experimental protocols were reviewed and approved by the ethical committee of the Maxima Medical Center, Veldhoven, The Netherlands.Informed consent was obtained from all participants. All participants in the study described were subjected to oral glucose tolerance test (GTT) based on an elevated risk for pregnancy diabetes, as established from clinical anamnesis and were sent in by either a gynaecologist or an obstetrician. In the first part of the study, blood was drawn from 30 subjects. Blood was collected in a lithium-heparin tube (BD Vacutainer, 367374). In addition, glucose concentration was determined by POC analysis (capillary whole blood from fingerstick, Roche Accuchek Inform II). All tubes were included in routine laboratory practice after phlebotomy and sample tracking was performed to gain insight in turn-around-time (TAT). TAT was defined as the delay between POC analysis and measurement of glucose in the laboratory. In the second part, blood was drawn from pregnant women that were subjected to a 75 gram GTT (n = 50). Blood was collected in lithium-heparin (BD Vacutainer, 367374) and NaF-EDTA-citrate (Terumo Venosafe VF-052SFC) tubes. In addition, glucose concentration was determined by POC analysis. Optimized laboratory conditions, defined as an analysis protocol according to the guidelines described for diabetes analysis^10^, were used for glucose analysis. In short, optimal laboratory conditions are achieved if one of the following three protocols are used: 1) STAT analysis; immediate separation of cells and plasma 2) cooling phlebotomized blood in an ice-water slurry and 3) the use of an immediate glycolysis inhibitor, such as citrate. Here, we employed protocols number 1 (STAT protocol) and 3 (feasible protocol), as protocol 2 was previously found to be sub-optimal^16^. All lithium-heparin tubes were centrifuged (4400 g, 5 minutes) directly after phlebotomy. All NaF-EDTA-citrate tubes were centrifuged 60 minutes after phlebotomy. Plasma was isolated immediately after centrifugation, kept on ice until storage, and subsequently stored at −80° Celcius until further analysis.

### Laboratory evaluation

POC glucose analysis was performed using a Roche Accuchek Inform II system. Plasma glucose concentration was determined on an automated Roche Cobas C501 analyzer (GLUC3 - Roche Diagnostics). HbA1c was determined by HPLC analysis (Menarini 8160, IFCC aligned). Fructosamine was determined on an automated Roche Cobas C502 analyzer (FRA - Roche Diagnostics). As per recommendation by the manufacturer, fructosamine concentration was corrected for total protein content, determined in the same sample (TP2 - Roche Diagnostics). All methods were controlled on a daily, regular basis and determined to be in control at the time of analysis.

### Clinical evaluation

Clinical evaluation of the 75 gram GTT was based on the 1999 WHO Guideline “Definition, Diagnosis and Classification of Diabetes Mellitus and its Complications”. Diagnosis of pregnancy diabetes was set if fasting glucose concentration was ≥6.1 mM or ≥7.8 mM after 120 minutes.

### Statistical analysis

Differences in glucose concentration determined by the routine, STAT and the feasible methods when compared to POC analysis at both time points were compared by student T test (to determine whether bias was constant over time). Deming regression analysis was performed using GraphPad Prism version 5.03 for Windows, with heparinized plasma (routine or STAT) as X-factor. Bland-Altman plots depict differences from heparin against the average of both reviewed methods (differences versus average). Clinical outcome (“yes” or “no” GDM) derived by POC, routine and optimized laboratory analysis were compared by Fisher exact test (GraphPad Software Inc., Accessed 12 April 2015). Correction of fructosamine for total protein content was performed using the following formula: Corrected fructosamine = measured fructosamine x 72/ total protein. Threshold for significance was set at 5%.

## Results

### Glucose concentration determined by POC vs. routine laboratory analysis

**Figure 1:**
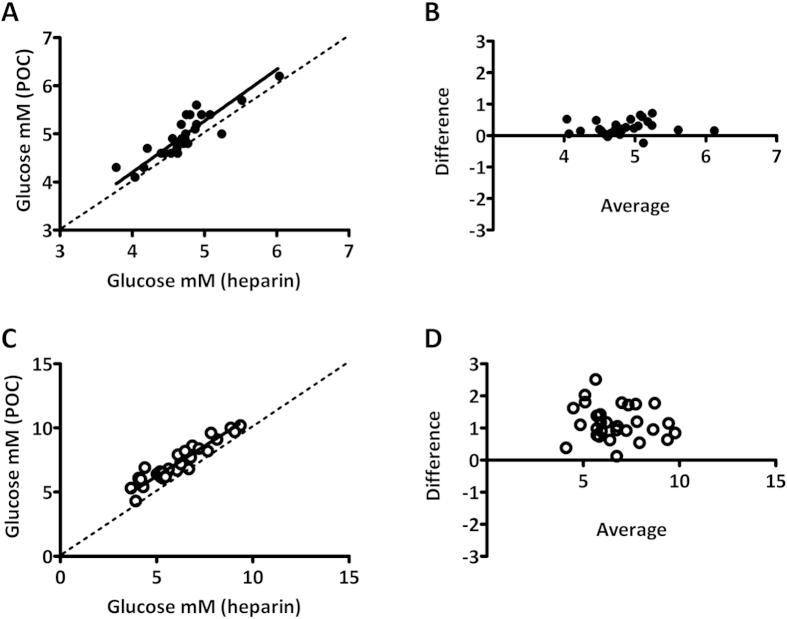
Comparison between POC and routine laboratory (heparin plasma) glucose measurement at t = 0 and t = 120 minutes of oral glucose tolerance test. (**A,C**) Deming regression plot for t = 0 and t = 120 minutes and (**B,D**) Bland-Altman difference plots for glucose concentration for t = 0 and t = 120 minutes. Plots depict difference in glucose concentration (POCT minus heparin) as a function of the average glucose concentration (POCT and heparin).

Glucose concentration determined by routine laboratory analysis correlated well with POC analysis (best fit 1.07, 95% CI 0.84 to 1.29 at t = 0 and best fit 0.95, 95% CI 0.81 to 1.08 at t = 120, respectively) but was lower at both timepoints. The average difference between methods was larger at t = 120 minutes (0.25 mM versus 1.17 mM at 0 and 120, respectively, p < 0.01, Fig. 1). A longer TAT at t = 120 minutes could possibly explain the difference^16^. However, median TAT was not longer (data not shown), making it unlikely that time explained the larger difference found between methods. To assess whether the difference between POC and routine laboratory glucose analysis was due to *in vitro* glycolysis, resulting in a drop in glucose concentration in the lithium-heparin tube, a second study was performed. This study was largely similar to the first study. However, in contrast to the first part, all heparin tubes were centrifuged immediately after phlebotomy (STAT analysis), eliminating bias due to *in vitro* glycolysis.

### Glucose concentration determined by POC vs. STAT laboratory analysis

**Figure 2:**
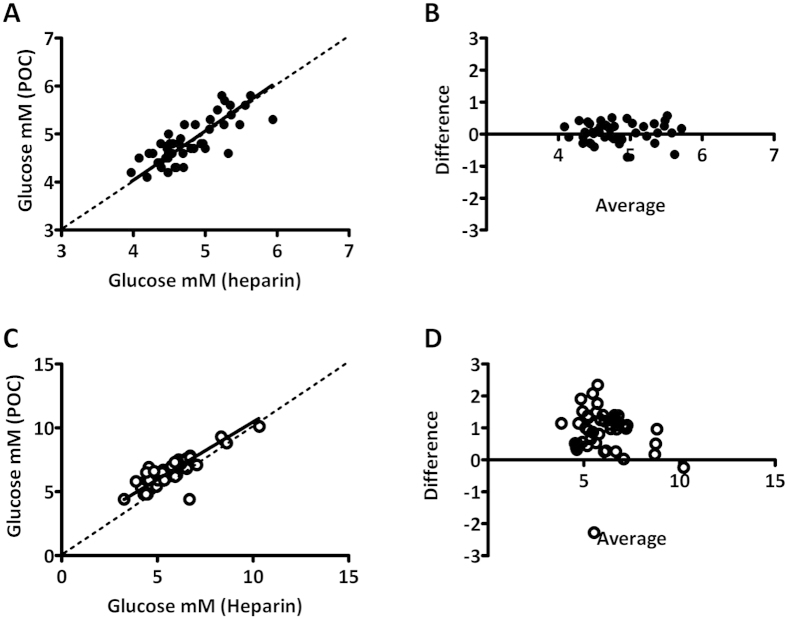
Comparison between POC and STAT laboratory (heparin plasma) glucose measurement at t = 0 and t = 120 minutes of oral glucose tolerance test. (**A,C**) Deming regression plot for t = 0 and t = 120 minutes and B,D) difference in glucose concentration between methods for t = 0 and t = 120 minutes. Plots depict difference in glucose concentration (POCT minus heparin) as a function of the average glucose concentration (POCT and heparin).

Similar to the comparison between POC and routine analysis, glucose levels correlated well between both methods (best fit 1.03, 95% CI 0.79 to 1.27 at t = 0 and best fit 0.90, 95% CI 0.73 to 1.07 at t = 120, respectively). Also here, average POC determined glucose concentration was higher when compared to STAT laboratory analysis. The difference between methods was larger at t = 120 minutes (0.06 mM versus 0.90 mM at 0 and 120, respectively, p < 0.01, Fig. 2). In conclusion; at both timepoints, the difference between by POC and laboratory analysis was approximately 0.3 mM less when analyzed by STAT analysis than when compared by routine laboratory analysis. Therefore, it is likely that TAT determines only a part (approximately 30%) of the total bias between POC and laboratory analysis.

### Glucose concentration determined by NaF-EDTA-citrate vs. STAT laboratory analysis

**Figure 3:**
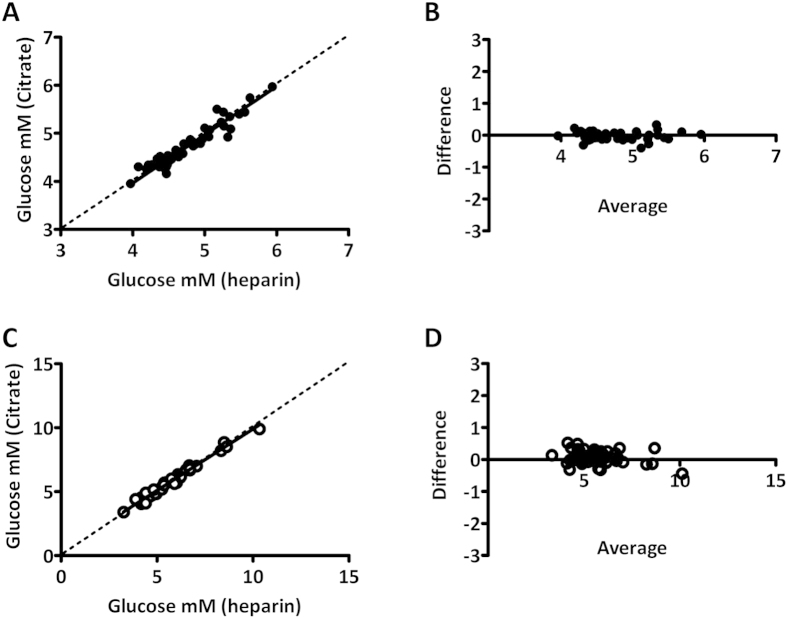
Comparison between STAT and feasible laboratory glucose measurements. (**A,C**) Deming regression plots for t = 0 and t = 120 minutes and B,D) Bland-Altman plots for glucose concentration for t = 0 and t = 120 minutes. Plots depict difference in glucose concentration (citrate minus heparin) as a function of the average glucose concentration (citrate and heparin).

**Figure 4:**
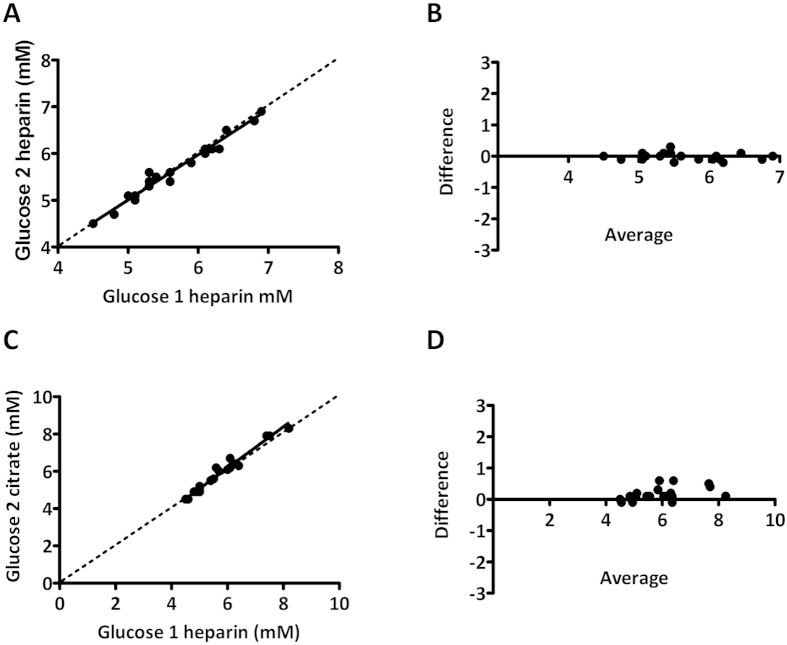
Comparison in glucose concentration determined between first and second tube drawn. (**A,C**) Deming regression plots and (**B,D**) Bland-Altman plots for glucose concentration. Plots depict difference in glucose concentration (second tube drawn minus first tube drawn) as a function of the average glucose concentration (second and first tube drawn).

As routine laboratory analysis results in significant loss of glucose due to *in vitro* glycolysis, STAT laboratory analysis is extremely laborious, and POC analysis resulted insignificant and variable bias, we have looked at an alternate approach, based on a feasible laboratory protocol. In the feasible protocol, citrated phlebotomy tubes were used, with a TAT of 60 minutes. Glucose concentrations determined by STAT laboratory analysis and determined using the feasible protocol correlated extremely well at both 0 and 120 minutes of GTT (Fig. 3). No significant slope was found at t = 0 (best fit 1.01, 95% CI 0.91 to 1.10) or t = 120 (best fit 0.96, 95% CI 0.92 to 1.01). In addition, no significant bias was found between methods at t = 0 (best fit −0.03, 95% CI −0.07 to 0.00) although a small but significant bias was found at t = 120 (best fit 0.06 mM 95% CI 0.00 to 0.13). The small bias found between both tube types may in part be due to the order of draw, measurement uncertainty and other environmental factors. Retrospective analysis of our previously published data^16^ where heparin control tubes were drawn prior to citrated tubes (after which both were centrifuged according to a STAT protocol) revealed similar bias and variation. Also, we found that a duplicate heparin draw showed less bias and variation (Fig. 4). The lower bias/variation in the duplicate heparin draw is most likely due to a similar loss in glucose in both tubes during the processing steps, for example during centrifugation. In conclusion, the use of citrated phlebotomy material with a TAT of 60 minutes enables a feasible GTT protocol, that yields results that are virtually identical to WHO recommended STAT protocol based on heparinized plasma analysis.

### Clinical evaluation

As expected from the glucose concentration measurements, POC analysis resulted in more GDM diagnoses (total of 11 subjects) when compared to routine laboratory conditions (total of 6 subjects). In All 5 subjects that had a different diagnosis in different methods, this was based on the timepoint 120 minutes (17%, p < 0.01). None of the subjects that were diagnosed with GDM by routine laboratory conditions were classified as non-GDM by POC analysis. Although POC analysis also resulted in more GDM diagnoses (total of 5 subjects) when compared to STAT laboratory conditions (total of 4 subjects), the difference was much less pronounced; no subjects were different at timepoint 0 minutes, and only 1 subject was misclassified as GDM at timepoint 120 minutes (2%, p = ns). None of the subjects that were diagnosed with GDM by routine laboratory conditions were classified as non-GDM by POC analysis. Clinical evaluation revealed that the protocol using citrated phlebotomy tubes resulted in the same number of GDM diagnoses when compared to STAT laboratory conditions. Out of 50 subjects, all women that had a conclusion “no GDM” using the STAT laboratory protocol were confirmed using the feasible protocol. Furthermore, 5 women were classified as GDM by both the STAT and feasible protocol.

### Secondary diabetes parameters

**Figure 5:**
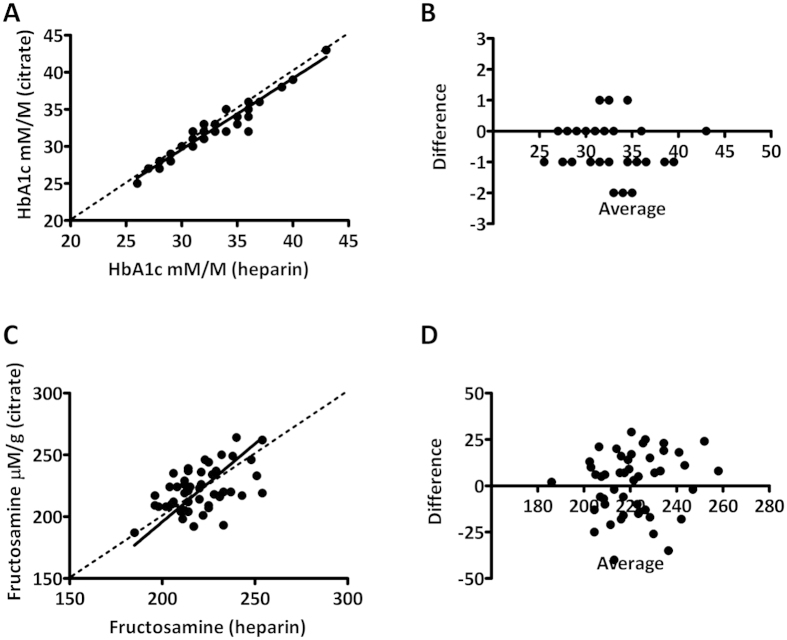
Comparison between STAT and feasible laboratory measurements for HbA1c and fructosamine. (**A,B**) Deming regression and Bland-Altman plots for HbA1c and (**B,D**) Deming regression and Bland-Altman plots for fructosamine. Bland-Altman plots depict differences in HbA1c and fructosamine (citrate minus heparin) as a function of the average concentration (citrate and heparin).

In addition to glucose, other parameters such as glycated haemoglobin (HbA1c) and fructosamine have been suggested in the context of diabetes screening and diagnosis. In 2011, HbA1c was put forward by the World Health Organization as a diagnostic parameter in the context of diabetes. We have compared HbA1c measurements in citrated whole blood compared to heparinized whole blood (Fig. 5). HbA1c levels were highly correlated, with a maximum deviation of 2 mM/M. No significant slope was found (best fit 0.96, 95% CI 0.89 to 1.03) between methods. In addition, no significant offset was found between methods (best fit 0.84, 95% CI −1.6 to 3.24). From these data we conclude that heparinized and citrated phlebotomy tubes can be used interchangeably for HbA1c analysis. In addition to HbA1c, we also have measured fructosamine in citrated and heparinized plasma. Fructosamine concentrations correlated well between methods as no significant offset was found (best fit1.26, 95% CI 0.60 to 1.93) between methods. In addition, no significant bias was found between methods (best fit −57, 95% CI −204 to 90). However, due to the large intra-individual variation in fructosamine concentration found between methods, citrated and heparinized plasma cannot be used interchangeably.

### Conclusions

POC glucose analysis has been opted to replace venous glucose analysis in the context of diabetes screening, especially under conditions where STAT clinical chemistry laboratory analysis is difficult or impossible. There has been a continuous debate on whether the higher intrinsic variation would result in a large number of misdiagnosis. Although this might be true, this argument could also hold for clinical chemistry laboratory analysis when non-optimized laboratory conditions are employed. An often overlooked laboratory condition is the actual turn-around-time (TAT). TAT is a significant determinant in the context of glucose concentration analysis^16^. Prolonged TAT results in significant loss of glucose due to ongoing glycolysis. Guidelines have been set up to ensure that *in vitro* glycolysis is kept to a minimum. The latest guideline^10^ states that, for correct analysis of glucose, one of the following protocols can be followed: 1) STAT analysis (immediate separation of cells and plasma) 2) cooling phlebotomized blood in an ice-water slurry and 3) the use of an immediate glycolysis inhibitor. Often, these protocols are adhered to only during the setup of clinical guidelines. For example, in the latest guideline for pregnancy diabetes^1^, blood was collected in standard sodium-fluoride tubes, but kept in an ice/water slurry, centrifuged in a pre-chilled centrifuge, followed by subsequent isolation and processing of the plasma. Under normal (routine) laboratory conditions, especially for small or satellite laboratories, these protocols are often impossible to adhere to. When exploring POC analysis as a replacement method, it is important to determine to what extend POC deviates from the optimal condition as well as to what extend the normal (routine) laboratory conditions deviate from the optimal condition. Here, we describe the direct comparison of POC glucose analysis to routine and optimized (STAT) laboratory conditions.

We have found that POC analysis classified more women as having GDM when compared to routine laboratory analysis. Also, we have also found that there was a large variation in TAT of tubes subjected to routine laboratory analysis, and that 10 out of 58 venous glucose measurements were delayed by more than 60 minutes before analysis. The TAT found in the current study is in line with the average TAT in Dutch clinical chemistry laboratories as we have found that it exceeds 60 minutes in more than 50% of laboratories^17^. In a different study^16^, we have found that glucose concentrations drop approximately 0.2 mM/h in lithium-heparin tubes, and that large differences exist between subjects. Given this, it is likely that the higher number of GDM diagnosis is not only due to overestimation by POC analysis but also due to underestimation by routine laboratory analysis. In the second part, we have compared POC and STAT chemistry glucose analysis. As expected, the differences in glucose levels between methods were much smaller as were the number of differentially classified cases. More specifically, POC analysis misclassified only 1 out of 50 cases (sensitivity 100%, specificity 98%). These data prove that the majority of GDM cases found by POC analysis are not solely due to the positive bias in the POC derived glucose measurement, but surely also due to a negative bias in the routine laboratory analysis protocol. Furthermore, these data also show that even though substantial bias exists between glucose concentration determined by POC and STAT laboratory analysis, POC analysis is a highly sensitive and specific method and is able to detect the vast majority of GDM cases when correct cut off values are used. From these data, we conclude that POC analysis is a viable alternative for STAT laboratory analysis, probably outperforming routine laboratory analysis in situations when a short TAT cannot be guaranteed.

Some might argue that a 2% false discovery rate is too high and that a large variability exists in the performance of POC glucose analyzers. From the comparison between POC, routine and STAT laboratory analysis, it became apparent that the deviation in glucose concentration was different when determined at t = 0 and t = 120 minutes. As TAT was essentially 0 in our second experiment, the positive bias could not be explained by ongoing glycolysis in the phlebotomy tube. An alternate hypothesis could be a matrix effect (plasma versus whole blood) and/or a time dependent difference in the glucose concentration of the pool sampled^8^. If the difference between methods could be explained by a matrix effect alone, one would expect the difference to be similar at timepoint 0 and 120 minutes. The glucose tolerance test induces a high concentration of glucose in the vascular compartment and a gradient between the vascular and interstitial compartment. In time, this glucose concentration will equilibrate with the surrounding interstitial fluid. Under fasting conditions, the time lag for glucose to enter the interstitial space is approximately 5 to 6 minutes in healthy subjects and below 10 minutes in type 1 diabetics^18^,^19^. In healthy subjects, venous glucose concentration peaks at approximately 1 hour and returns to baseline after approximately 2 hours after an oral glucose load. In subjects with impaired glucose tolerance, peak levels are higher, and the return to baseline is delayed. It could therefore be that the difference between methods is also partly explained by sample pool concentration differences. Also, one can clearly see that large inter-individual exist in terms of difference between POC and venous glucose concentration. For example, when comparing POC to STAT glucose analysis, some of the subjects showed differences in excess of 2 mM. These individual differences may in part be due to their individual (glycemic) control, resulting in prolonged lag time for glucose to enter and leave the interstitial fluid compartment. As previously mentioned, average lag time is below 10 minutes for healthy individuals. However, a wide range is seen between subjects, and individual lag times may be as long as 45 minutes^20^. Different lag times may result in augmented differences between both compartments in a time dependent manner. Also, one has to take into account that all subjects were given the same amount of glucose (75 grams), independent of body mass and composition, which would proportionally affect the imbalance between venous and interstitial fluid glucose concentration.

Given the larger intra-assay variation and the unexplainable and unpredictable bias in the POC analysis at t = 120 minutes, we have also performed an experiment comparing a feasible laboratory protocol (based on citrated phlebotomy material) to STAT laboratory analysis. In the feasible protocol, we have used commercially available tubes and kept a TAT of 60 minutes before centrifugation of the material. We have found that this protocol yielded identical results when compared to STAT laboratory analysis in terms of glucose concentration as well as clinical outcome. None of the 50 women studied were misclassified in terms of GDM. Recently, it was shown that the tubes we used were not only stable within a 1 hour time window, but stabilized glucose concentrations for a period of at least 24 hours^21^. Combined, these data show that citrated phlebotomy tubes enable a feasible method for pregnancy diabetes testing, with results identical to the results obtained with the WHO recommended protocol. In addition, other parameters that may be of interest in diabetes screening and follow up were tested. In contrast to HbA1c, the position of fructosamine in diabetes screening is much less evident^22^,^23^. However, it has been postulated that in patients who require frequent blood transfusion, use certain types of medication^24^ or in the presence of Hb variants and/or abnormalities in RBC turnover^25^, HbA1c may not be a reliable marker for glycemic status. Fructosamine may also reflect the status of blood glucose more rapidly (2–3 weeks) than HbA1c (2–3 months)^26^. HbA1c levels were highly similar and almost identical when measured in citrated and heparinized tubes. Therefore, we conclude that both tube types may be used interchangeably in the follow up or diagnosis of diabetes. Average fructosamine levels were similar but large intra-individual differences found when comparing data from heparinized and citrated plasma. Given the relatively small intra-individual biological variation it may not be possible to use data generated in both tubes interchangeably^27^. Given the small sample size of the current study, we did not focus on the usability of either HbA1c or fructosamine in the diagnosis of pregnancy diabetes.

Here, we show that pre-analytical variables such as method, TAT and tube type play an important role in the diagnostic path for pregnancy diabetes. Bias may be introduced by inadequate handling of blood specimens, for example due to ongoing glycolysis, but also due to sample pool selection (plasma versus whole blood). Also, it is important to keep in mind that not all analyzers are equal. This is especially true for POC meters. POC meter specific bias may be as large as 10%^28^ and lot-to-lot reagent dependent. The same, although to a much lesser extent, holds for laboratory chemistry analyzers. Bias should be established, adjusted if needed and monitored regularly by interlaboratory proficiency testing before one can use the meter appropriately.

In conclusion, we have found that comparison of POC glucose analysis to laboratory analysis may be flawed if non-optimized laboratory conditions are used, due to *in vitro* glycolysis. Furthermore, we show that POC analysis is associated with a substantial positive bias concerning absolute glucose concentration, but enables a reliable diagnostic tool for pregnancy diabetes screening if the correct cut-off values are used. Furthermore, we provide a WHO recommendation compatible, but feasible laboratory protocol for pregnancy diabetes screening, based on NaF-EDTA-citrate containing phlebotomy tubes with a turn-around-time between phlebotomy and centrifugation of 60 minutes.

## Additional Information

**How to cite this article**: Berg, S. A.A. *et al.* Pregnancy diabetes: A comparison of diagnostic protocols based on point-of-care, routine and optimized laboratory conditions. *Sci. Rep.* **5**, 16302; doi: 10.1038/srep16302 (2015).
